# Effects of light-emitting diode supplementary lighting on the winter growth of greenhouse plants in the Yangtze River Delta of China

**DOI:** 10.1186/s40529-015-0117-3

**Published:** 2016-01-18

**Authors:** Xue Li, Wei Lu, Guyue Hu, Xiao Chan Wang, Yu Zhang, Guo Xiang Sun, Zhichao Fang

**Affiliations:** 1 College of Engineering, Nanjing Agricultural University, Nanjing, Jiangsu People’s Republic of China; 2 Jiangsu Province Engineering Lab for Modern Intelligent Facilities of Agriculture Technology & Equipment, Nanjing, Jiangsu People’s Republic of China

**Keywords:** LED, Supplementary lighting, Greenhouse, Winter, Pepper

## Abstract

**Background:**

The winter in the Yangtze River Delta area of China involves more than 1 month of continuous low temperature and poor light (CLTL) weather conditions, which impacts horticultural production in an unheated greenhouse; however, few greenhouses in this area are currently equipped with a heating device. The low-cost and long-living light-emitting diode (LED) was used as an artificial light source to explore the effects of supplementary lighting during the dark period in CLTL winter on the vegetative characteristics, early yield, and physiology of flowering for pepper plants grown in a greenhouse without heating. Two LED lighting sets were employed with different light source to provide 65 μmol m^−2^ s^−1^ at night: (1) LED-A: red LEDs (R, peak wavelength 660 nm) and blue LEDs (B, peak wavelength 460 nm) with an R:B ratio of 6:3; and (2) LED-B: R and B LEDs at an R:B ratio of 8:1. Plants growth parameters and chlorophyll fluorescence characteristics were compared between lighting treatments and the control group.

**Results:**

Plants’ yield and photosynthesis ability were improved by LED-A. Pepper grown under the LED-A1 strategy showed a 303.3 % greater fresh weight of fruits and a 501.3 % greater dry mass compared with the control group. Plant leaves under LED-A1 showed maximum efficiency of the light quantum yield of PSII, electron transfer rate, and the proportion of the open fraction of PSII centers, with values 113.70, 114.34, and 211.65 % higher than those of the control group, respectively, and showed the lowest rate constant of thermal energy dissipation of all groups. LED-B was beneficial to the plant height and stems diameter of the pepper plants more than LED-A.

**Conclusions:**

These results can serve as a guide for environment control and for realizing low energy consumption for products grown in a greenhouse in the winter in Southern China.

## Background

Light and temperature are the most important environmental factors affecting plant survival, dry mass distribution, and crop yield (Janda et al. 2014; Zoratti et al. 2014). Light is the direct energy source for plant photosynthesis to determine the distribution of organic materials, the morphogenesis of plant and subsequent yield (Blom and Ingratta 1984; Fan et al. 2008). The temperature state can affect the activity of enzymes in plant cells to exert physiological changes (Chen and Tang 2013). Because of this close relationship between temperature and physiological reactions, temperature and lighting cues provide vital information for plants to assure optimal development (Franklin et al. 2014). Under the condition of light with a low red/far-red (R/FR) ratio and a comfortable temperature, the plant leaf area and dry mass are reduced (Patel et al. 2013); however, at lower temperatures, lighting with a low R/FR ratio can significantly promote the plant-soluble sugar content and the expression of C-repeat-binding factor, a regulator of cold-acclimation genes (Franklin and Whitelam 2007). Furthermore, Marcelis (1993a, b) found that plant fruits dry mass were improved with increasing irradiance and decreasing temperature, demonstrating that when plants are in a relatively low temperature environment (or suboptimal temperature), adjusting the lighting condition exerts positive effects on plant production.

A light-emitting diode (LED) can emit the monochromatic light required for plant growth. The combination of red LEDs with blue LEDs can form a spectral absorption peak suitable for plant photosynthesis and morphogenesis with an 80–90 % light energy utilization rate and a remarkable energy-saving effect (Yang. 2008). Some reports have indicated that the use of a single red or blue LED light source or their combination could improve the efficiency of photosynthesis to promote plant production and regulate morphogenesis (Barta et al. 1992; Fang and Jao 1996; Nhut et al. 2003; Ding et al. 2005; Xiao et al. 2013; Shen et al. 2014).

In the Yangtze River Delta region of southern China, the low temperature and poor light (CLTL) environment always lasts for more than 1 month in winter (Wu 2011). Moreover, greenhouses in this area are scarcely heated owing to the high energy consumption and costs. Consequently, this suboptimal weather in southern China is quite detrimental to plant growth and production. Most of the research conducted in this area has focused on seedling growth in response to CLTL conditions or has examined the influence of only a single factor on plant growth in order to select plants that are resistant to the harsh temperature conditions (Wang et al. 2001; Ren et al. 2002; Hu and Yu 2003). Little studies have been conducted to study how to improve the production of flowering plants grown in greenhouse under the CLTL conditions. Thus, the aim of this study was to evaluate the effects of LED supplementation on flowering plants grown in a greenhouse without heating equipment during the dark period in winter. These results should serve as a guide for environmental control and for achieving low energy consumption with high productivity in plants grown in greenhouses in the winter in southern China.

## Methods

### Plant material and experimental design

Experiments were conducted from December 2014 to January 2015 in a greenhouse located in the College of Engineering in Nanjing Agricultural University (Nanjing, Jiangsu Province, China, 118°46′ N, 32°03′ E), which is characterized by a sub-tropical monsoon climate. The greenhouse is equipped with internal and external shading, skylights, a drip irrigation system, and a spray system, which are all computer-controlled. The Eco-Watch ecological environment monitoring system (Dynamax, USA) was used to monitor the micro-environmental parameters inside the greenhouse. Pepper (*Capsicum frutescens* L., Sujiao No. 5) was chosen as the experimental material. The seed of pepper were sown in nutrient soil (Galaku Pty Ltd., Australia) on September 10, 2014 and transplanted into plots after the fourth true leaf was fully mature. The medium was a coconut shell (Galaku Pty, Ltd.). The Yamazaki nutrient (Table 1) solution was used to supply nutrients for pepper growth. Chlorophyll fluorescence parameters were measured with the Mini-Pam II system (Walz, Germany), quantum photon density and light homogeneity were tested by a PAR sensor equipped by Mini-Pam II.

**Table 1:** Elements composition of Yamazaki nutrient in 1 L deionized water

Elements composition	Dosage (mg L^−1^)
Ca(NO_3_)_2_·4H_2_O	354
KNO_3_	607
NH_4_H_2_PO_4_	96
MgSO_4_·7H_2_O	185
Na_2_Fe-EDTA	25
H_3_BO_3_	2.13
MnSO_4_·4H_2_O	2.86
ZnSO_4_·7H_2_O	0.22
CuSO_4_·5H_2_O	0.08
(NH_4_)_6_MO_7_O_2_·4H_2_O	0.02

The LED lighting sets were established for each plant individually. Each light set consisted of 144 red (R, peak wavelength 660 nm) or blue (B, peak wavelength 460 nm) LEDs with ultra-high brightness at an R:B ratio of 6:3 (LED-A group) or 8:1 (LED-B group) (Hogewoning et al. 2010). The lighting area was 555 × 22.8 mm. The plant photosynthetic quantum flux density, measured 15 cm above the plants, was 65 μmol m^−2^ s^−1^.

Plants in anthesis with similar growth trends were chosen for the experimental treatment. Supplementary lighting began on December 1, 2014 and finished on January 15, 2015. The humidity arranged from 60 to 70 %. Temperature was the environment temperature as well as the light. The experiment included seven treatments: six experimental groups and the control group, with six plants in per treatment. The control group was plants grown without light supplementation at night. The supplementary lighting duration and light source for each treatment are given in Table 2.

**Table 2:** The light quality and light supplementation arrangement

Light source	R/B ratio	Lighting duration	Serial number
LED-A	6:3	18:00–00:00 (6 h)	LED-A1
	6:3	18:00–22:00 (4 h)	LED-A2
	6:3	18:00–20:00 (2 h)	LED-A3
LED-B	8:1	18:00–00:00 (6 h)	LED-B1
	8:1	18:00–22:00 (4 h)	LED-B2
	8:1	18:00–20:00 (2 h)	LED-B3
–	–	–	Control group

### Parameter measurements

The greenhouse environmental parameters were collected by the Eco-Watch system at 15-min intervals. All pepper parameters were measured after 2 weeks of light supplementation at night period. The chlorophyll fluorescence parameters of the pepper plants were tested every 7 days, with healthy canopy leaves chosen for measurement, ensuring that each measure was taken at the same location as much as possible. The chlorophyll fluorescence parameters would be measured for 5 weeks in all. The actinic light intensity was four and the SAT-Plus intensity was 10, the duration of induction curve was 20 s with the length of 12. The measuring time was from 17:00–21:00 after 25 min of dark adaptation for the leaves, and three leaves were tested of each plant with the averages used for statistical analysis. Three plants were randomly tested in each treatment. At the end of the experiment, the following vegetative characteristics of the plants were measured: plant height, stem diameter, and plant canopy width. The dry mass of the roots, stems, leaves, and fruits were also recorded.

### Statistical analysis

Excel 2010 (Chinese version) and Origin 7.5 (Chinese version) were used for data processing and graph generation. Analysis of variance (ANOVA) was used to evaluate statistical significance among groups, and the Duncan method was used for multiple comparisons evaluated at p < 0.05 or p < 0.01 with SPSS 17.0 (Chinese version).

## Results

### Environmental parameters in the greenhouse during winter

**Fig. 1:**
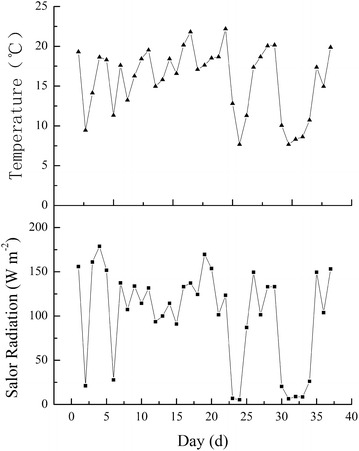
Daily microclimate conditions (air temperature and solar radiation) inside the greenhouse. The data were the average of temperature and solar radiation from 6:00 to 17:00

Figure 1 showed the temperature and solar radiation fluctuations during daytime hours in the greenhouse. The daily minimum temperature was 7.67 °C, the average temperature was 15.8 °C, and the highest temperature was 22.18 °C. With respect to solar radiation, the minimum value was 5.35 W m^−2^, the average value was 101.5 W m^−2^, and the maximum value was 178.86 W m^−2^, indicating that the peppers grown in the greenhouse were in a CLTL environment.

### Plant growth and development in cltl with supplementary led lighting during dark periods in winter

#### Impacts of LED supplementary lighting on plant vegetative characteristics during the dark period

**Fig. 2:**
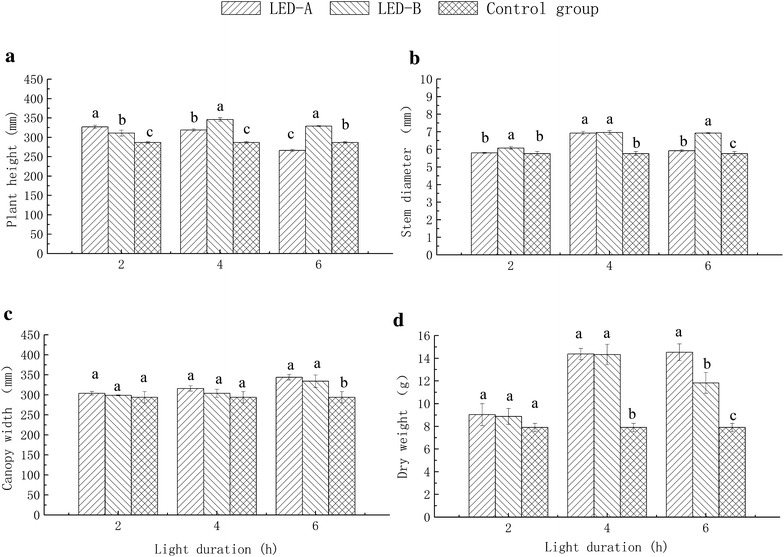
Vegetative characteristics and dry mass of peppers grown under LED light supplementation during dark period. *Different letters* indicate a significant difference at p < 0.05. The R:B ratios of LED-A and LED-B were 6:3 and 8:1, respectively, and the control group received no light. **a** illustrates the plant height under different lighting treatments; **b** illustrates the stem diameter of plants under different lighting treatments; **c** explains the canopy width of plants under different lighting treatments; **d** explains the whole dry weight of plants under different lighting treatments

When the lighting was applied for 2 h during the dark period, plants grown under the LED-A treatment showed the highest plant height, which was 13.94 % higher than that of the control group, representing a significant difference (p < 0.05; Fig. 2a). The stem diameter under the LED-B treatment was significantly higher than that of the control with an increase of 5.56 % (p < 0.05; Fig. 2b). No significant difference in plant canopy width or plant dry mass was observed among the groups.

When the LED lighting was applied for 4 h, the plants grown under the LED-B treatment showed the highest plant height and stem diameter, which were 20.44 and 20.83 %, respectively, greater than those of the control group (Fig. 2a, b). Plants grown under LED-A showed significantly increased plant dry mass of 81.86 % compared to the control group (p < 0.05; Fig. 2d). There was no significant difference among the groups on plant canopy width.

When the lighting was applied for 6 h in CLTL winter, the plants height under the LED-B treatment was significantly higher than that of the LED-A and control groups (p < 0.05), with a 14.63 % increase compared to the control (Fig. 2a). The stem diameter of plants grown under the LED-B treatment was significantly greater than that of plants under the LED-A treatment, and was increased by 20.31 % compared to the control (Fig. 2b). The plant canopy width under LED-A was 17.01 % greater than that of the control (Fig. 2c; p < 0.05). There was no significant difference in plant canopy width between the LED-A and LED-B groups. Plants grown under the LED-A treatment showed the largest plant dry mass, which was 83.96 % greater than that of the control, which showed the lowest level of all groups; the difference among groups was statistically significant (p < 0.05, Fig. 2d).

#### Effects of different lighting strategies on early fruit yield and plant dry mass distribution

The dry mass distribution ratio can reflect the effects of lighting on the dry mass distribution to all of the pepper plant organs, whereas the shoot-to-root ratio reflects the effects on the dry mass distribution of the under-ground parts relative to the over-ground parts. As shown in Table 3, the root growth of plants under LED-B was higher than the LED-A and the control, while the stem growth of plants grown under LED-B was significant greater than the control as well as the LED-A (p < 0.05). Plants grown under LED-A1 showed the lowest leaf dry mass ratio, which was 24.32 % lower than that of the control. Compared with the control, plants grown under the LED-B treatment showed no significant difference in the leaf dry mass ratio and fruit dry mass ratio, whereas lighting strategy LED-A1 appeared to be the most conducive for dry weight accumulation of fruits, with a 200.00 % increase compared to that in the control groups. There was no significant difference in the shoot-to-root ratio among treatments excluded the LED-B2.

**Table 3:** Dry mass distribution ratio of pepper under different lighting strategies

Lighting strategy	Dry mass distribution ratio of pepper				
	Root	Stem	Leaf	Fruit	Shoot/Root
LED-A1	0.14 ± 0.01^ab^	0.25 ± 0.01^e^	0.28 ± 0.02^d^	0.33 ± 0.02^a^	6.42 ± 1.07^ab^
LED-B1	0.16 ± 0.01^a^	0.35 ± 0.02^bc^	0.35 ± 0.01^b^	0.14 ± 0.02^c^	5.27 ± 0.40^b^
LED-A2	0.14 ± 0.02^ab^	0.30 ± 0.01^d^	0.30 ± 0.01^c^	0.26 ± 0.03^b^	6.38 ± 0.86^ab^
LED-B2	0.11 ± 0.02^b^	0.39 ± 0.01^a^	0.38 ± 0.01^a^	0.12 ± 0.01^cd^	7.67 ± 1.19^a^
LED-A3	0.16 ± 0.02^a^	0.35 ± 0.01^bc^	0.37 ± 0.01^a^	0.12 ± 0.01^cd^	5.27 ± 0.21^b^
LED-B3	0.15 ± 0.02^a^	0.37 ± 0.01^a^	0.38 ± 0.01^a^	0.10 ± 0.00^d^	5.73 ± 0.83^b^
Control group	0.16 ± 0.01^a^	0.35 ± 0.01^bc^	0.37 ± 0.01^a^	0.11 ± 0.01^cd^	5.13 ± 0.21^b^

Data are the mean ± SD of each group. Different letters in same column indicate a significant difference at p < 0.05

As shown in Table 4, LED-A promoted the greatest yield of plants in CLTL conditions of winter. In addition, the fruit fresh and dry weights per plant grown under LED-A were significantly higher than those of the LED-B and control groups, with increases of 238.02 and 305.06 % compared to the control, respectively. Together, these results demonstrate that supplementary lighting augmented plant dry mass production (Fig. 2d), and promoted partitioning of the dry mass to the fruit (Tables 3 and 4). Moreover, LED-A supplementation could reduce the distribution of the dry mass of plants to the stems and leaves, resulting in an effective increase in fruit dry mass accumulation to ultimately increase plant yield. Specifically, plants grown under the LED-A1 strategy showed the highest fruit yield per plant, which was significantly higher than that of control group with a 303.33 and 501.27 % increase in fresh weight and dry mass of fruits, respectively.

**Table 4:** Early fruits yield of pepper under different LED lighting strategies

Lighting strategy		Average fresh weight (g plant^−1^)	Average dry weight (g plant^−1^)	Average fruit number
LED-A	LED-A1	32.67 ± 2.00^a^	4.75 ± 0.38^a^	9.3 ± 0.7^a^
	LED-A2	26.50 ± 1.68^ab^	3.79 ± 0.52^b^	6.0 ± 0.7^b^
	LED-A3	22.98 ± 4.44^bc^	1.05 ± 0.15^d^	6.5 ± 0.7^b^
LED-B	LED-B1	16.15 ± 5.85 ^cd^	1.68 ± 0.50^c^	6.0 ± 0.7^b^
	LED-B2	14.4 ± 7.10^de^	1.72 ± 0.20^c^	5.6 ± 0.7^b^
	LED-B3	8.3 ± 0.4^e^	0.92 ± 0.08^d^	6.3 ± 0.7^b^
Control group		8.1 ± 1.40^e^	0.79 ± 0.04^d^	5.7 ± 0.7^b^

Data are the mean ± SD of each group. Different letters in same column indicate a significant difference at p < 0.05

#### Chlorophyll fluorescence characteristics under different lighting strategies during the dark period

**Fig. 3:**
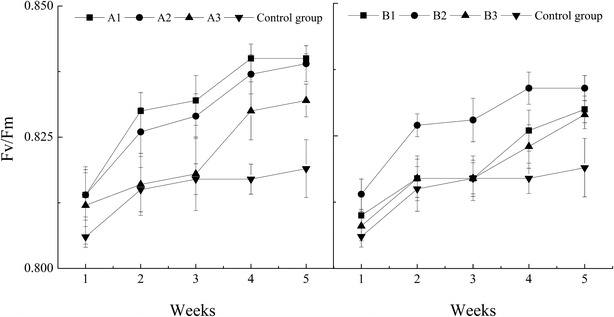
Fv/Fm curve of pepper leaves for different weeks under different light supplementation strategies at night. Data are the mean ± SD of each group

The Fv/Fm ratio is the potential maximum photosynthetic ability, which is steadily maintained at 0.8–0.85 in healthy plants, and decreases in stress conditions; thus, Fv/Fm is an importance index for studying the impact of various environmental stresses on plant photosynthesis (Björkman and Demmig 1987; Pathre and Shirke 2003). Table 5 shows that the CLTL environment in winter did not destroy the photosynthetic function of peppers plants, given that the Fv/Fm ratio remained basically stable at 0.8–0.85. Furthermore, the trends in Fv/Fm dynamics were similar among the LED-A, LED-B, and control group (Fig. 3), showing an increase trend, indicating that all of the pepper plants became increasingly stronger and healthier and that the initial light energy conversion efficiency was maintained. Peppers had the highest Fv/Fm under the LED-A1 condition (Table 5).

**Table 5:** Effects of LED supplementation on chlorophyll fluorescence parameters of pepper

Lighting strategy	Y(II)	ETR	qL	NPQ	Fv/Fm
LED-A1	0.312 ± 0.090^a^	11.81 ± 3.51^a^	0.321 ± 0.130^a^	1.419 ± 0.256^d^	0.831 ± 0.014^a^
LED-B1	0.259 ± 0.065^b^	9.89 ± 2.59^b^	0.216 ± 0.085^b^	1.605 ± 0.210^ab^	0.820 ± 0.013^cd^
LED-A2	0.201 ± 0.066^c^	7.02 ± 2.96^c^	0.131 ± 0.072^c^	1.570 ± 0.249^bc^	0.829 ± 0.015^a^
LED-B2	0.176 ± 0.049^c^	7.40 ± 1.50^c^	0.135 ± 0.039^c^	1.700 ± 0.187^a^	0.827 ± 0.011^ab^
LED-A3	0.178 ± 0.073^c^	6.76 ± 2.81^c^	0.126 ± 0.073^c^	1.622 ± 0.183^ab^	0.821 ± 0.019^bc^
LED-B3	0.146 ± 0.053^d^	5.65 ± 1.93^d^	0.104 ± 0.047^c^	1.439 ± 0.270^d^	0.818 ± 0.012^cd^
Control group	0.146 ± 0.059^d^	5.51 ± 2.24^d^	0.103 ± 0.063^c^	1.479 ± 0.286^cd^	0.815 ± 0.012^d^

Data are the mean ± SD of each group; Different letters in same column indicate a significant difference at p < 0.05

The parameter Y(II) is used to directly estimate the efficiency of the actual captured energy of the PSII photosynthetic reaction center (Bilger and Björkman 1990), reflecting the amount of excitation energy reaching the PSII reaction center (Schreiber et al. 1995). The electron transfer rate (ETR) is used to reflect the photosynthesis rate under the actual light intensity (Kramer et al. 2004). As shown in Table 5, the Y(II) and ETR values in the experimental groups were higher than those in the control group. The Y(II) and ETR values of plants under LED-A1 strategy were significantly higher than those in the control group and other treatment groups (p < 0.05); the maximum values observed in the LED-A1 group were 113.70 and 114.34 % higher than the control group, respectively. This indicated that the LED-A1 strategy is the most helpful for improving the photoreaction rate of the PSII reaction center, which in turn promotes the electron transfer ability of the PSII to ultimately increase the assimilatory power (ATP and NADPH) in PSI and improves the dry mass accumulation of peppers.

The parameter qL reflects the proportion of light absorbed by the antenna pigment in PSII for the photochemical reaction (Kramer et al. 2004), and NPQ is proportional to the rate constant of thermal energy dissipation, which is related to the plant self-protection mechanism of the photosynthetic apparatus in a stressful environment (Zribi et al. 2009). Plants grown under the LED-A1 strategy had the highest qL, which was significantly higher (p < 0.05) than that of other treatments with a 211.65 % increase compared to the control. In addition, there was a significantly correlated relation among qL, Y(II) and ETR (p < 0.01), indicating that these parameters were to reflect the actual redox degree of photoelectrons in PSII. The NPQ of plants under the LED-B treatment was significantly higher than that of other groups; especially LED-B2 was the highest one with a 14.94 % increase compared to the control, indicating that the light absorbed by the plant’s antenna pigments was dissipated as heat to a greater extent in the LED-B light quality, and that the plants’ self-protection mechanisms of the photosynthetic apparatus in PSII was strengthened. The NPQ of plants under the LED-A1 strategy was the lowest, significant lower than the groups and with a 4.06 % increase compared to the control, indicating that more light could be used for the photochemical reaction under this condition (Table 5).

#### Effects of light quality and lighting duration on the chlorophyll fluorescence parameters in winter

As shown in Table 6, the light source of LEDs had a significant effect on Y(II), qL, Fv/Fm, and ETR values (p < 0.01). The lighting duration had extreme significant influence on all chlorophyll fluorescence parameters (p < 0.01). Light source and lighting duration had a significant impact on Fv/Fm and ETR (p < 0.05) and a highly significant effect on qL and NPQ (p < 0.01).

**Table 6:** Two-way ANOVA results (F-values) for effects of supplementary light source and duration on the chlorophyll fluorescence parameters of pepper plants

Source	Y(II)	QL	NPQ	Fv/Fm	ETR
Light source	20.128**	18.393**	2.274	10.089**	7.848**
Light duration	85.719**	104.381**	7.046**	7.986**	80.325**
Light source × Light duration	1.012	12.104**	15.821**	3.138*	4.604*

* and ** indicate significant difference at p < 0.05 and p < 0.01, respectively

## Discussion

China’s Yangtze River Delta region is the most concentrated area of horticulture cultivation in the country, and its economic benefits directly reflect the productivity level of horticulture cultivation of China. However, due to its geographical location and meteorological conditions, the area is subject to more than 1 month of a harsh CLTL environment in winter, with severe consequences for production in plants grown in a greenhouse without a heater. Therefore, studies focused on improving greenhouse production in winter in this region should be a priority on the basis of plant physiology combined with agricultural engineering technology theory. In this study, LED artificial supplementary lighting technology was exposed to pepper plants grown in a greenhouse under CLTL winter conditions during dark periods to detect the influences of different light supplementation strategies on the growth and development of pepper plants.

Hao and Papadopoulos (1999) found that plant dry mass production could be increased through lighting supplementation, and dry mass allocation was increased to the fruit and decreased to the stem. In this study, the total and fruits dry mass of plants with light supplementation was significantly higher than the control, however, though the stem dry mass ratio under LED-B was significant higher than the control but it was decreased under LED-A, plant height and stem diameter were higher under LED-B than under LED-A and control lighting conditions, the stem and leaf dry mass ratio of plants under the LED-B strategy were higher than those under LED-A and control conditions, indicating that red light plays an important role in promoting elongation of the plant stem and dry matter accumulation, in order to guide assimilation of the mass to the stem and leaf (Moe et al. 2002). Blue light facilitates leaf expansion and helps to assimilate products to the fruit (Chu et al. 1999; Ni et al. 2009). Thus, the stem dry mass ratio of plants under LED-A, in this study, was lower than the control. While, the pepper leaf width was slightly higher under LED-A than that under LED-B and the control, and the fruit dry mass ratio and yield were significantly higher under LED-A than those of LED-B plants.

The duration of supplementary lighting directly affects the photoperiod of the plant, which determines the duration of plant photosynthesis, thereby affecting carbohydrate metabolism and the absorption and transformation of nutrients (Jin et al.^2014^). In this study, plants under an LED-A1 and LED-B1 strategies with an 18-h photoperiod showed the largest fruit dry mass ratio as well as fruit fresh weight and dry mass per plant, followed by the LED-A2 and LED-B2 strategies with a 14-h photoperiod; the LED-A3 and LED-B3 treatments showed the lowest values with respect to productivity.

The photosynthesis ability of plant leaves is directly proportional to the R/B ratio of LEDs (Hogewoning et al. 2010). While the results of this study showed that both light source and light duration had a significant impact on ETR (can be used to evaluate the photosynthetic rate of plant leaves), qL (the open ratio of photochemical reaction in PSII), Fv/Fm and NPQ. Therefore, in this study the R/B ratio of the LEDs is only one aspect that contributes to photosynthetic ability. In addition, the Fv/Fm of plants under LED-A was higher than that under LED-B when the lighting duration was the same, and was significantly increased compared to that of the control group. These results suggest that when using supplementary lighting sources with different R/B ratios, an LED with a higher amount of blue would increase the Fv/Fm, which could improve the original capturing ability of light and enhance the health of peppers. However, the optimal R/B ratio requires further study as well as the duration of supplementary lighting.

## Conclusions

The results of the present study showed several benefits of LED supplementary lighting during the dark period for pepper plants grown in greenhouse in a CLTL environment. First, LED-A is particularly beneficial for promoting the production of peppers grown in the winter in a greenhouse without a heater, whereas LED-B is helpful to increase the height and stem of plants. Second, the photosynthesis ability of plants was influenced by both light source and the lighting duration of the supplementary lighting setting. In addition, a lighting setting with a high blue-to-red light ratio is more favorable for enhancing the pepper plant’s natural ability to capture light energy and can improve the photosynthetic ability of the plants as well.

## Abbreviations

CLTL: continuous low temperature and poor light
h: hour
PSII: photosystem II
Y(II): the light quantum yield of PSII
ETR: electron transfer rate
qL: fraction of PSII centers that are open
NPQ: rate constant of non-photochemical quenching
Fv/Fm: maximum photochemical efficiency of PSII
